# Endolymphatic hydrops in the unaffected ear of patients with unilateral Ménière’s disease

**DOI:** 10.1007/s00405-022-07412-9

**Published:** 2022-05-16

**Authors:** Carlos Guajardo-Vergara, Victor Suárez-Vega, Pablo Dominguez, Raquel Manrique-Huarte, Lorea Arbizu, Nicolás Pérez-Fernández

**Affiliations:** 1 Department of Otorhinolaryngology, Clínica Universidad de Navarra, Pamplona, Spain; 2 Department of Radiology, Clínica Universidad de Navarra, Madrid, Spain; 3 Department of Radiology, Clínica Universidad de Navarra, Pamplona, Spain; 4 Department of Otorhinolaryngology, Clínica Universidad de Navarra, Marquesado de Santa Marta 1, 28027 Madrid, Spain; 5 Escuela de Fonoaudiología, Universidad Austral de Chile, Sede Puerto Montt, Los Lagos, Chile; 6 Department of Otorhinolaryngology, Complejo Hospitalario de Navarra, Pamplona, Spain

**Keywords:** Endolymphatic hydrops, Ménière’s disease, Vestibular-evoked myogenic potentials, Magnetic resonance imaging

## Abstract

**Purpose:**

Current studies show that frequency tuning modification is a good marker for the detection of endolymphatic hydrops (EH) employing magnetic resonance imaging (MRI) in patients with Ménière’s disease (MD). The purpose of the present study is to analyze the auditory and vestibular function with audiometric and vestibular-evoked myogenic potentials (VEMP) responses, respectively, in both the affected and unaffected ears of patients with unilateral MD using MRI as diagnostic support for the degree of EH.

**Methods:**

We retrospectively reviewed the medical records of 76 consecutive patients with unilateral definite MD (age 55 (28–75); 39 women, 37 men). MRI was used through intravenous gadolinium administration, audiometry, and VEMPs. Functional tests were performed up to a week after the MRI. All were followed up one year after imaging utilizing clinical, auditory, and vestibular testing to rule out bilateral involvement.

**Results:**

In the unaffected ear, the mean pure-tone average is normal even in cases with hydrops and, for a similar severity of hydrops is significantly lower than in the affected ear. Significant differences for the amplitude of the response at 0.5 kHz, at 1 kHz between the affected and unaffected ears were found to be lower in the affected ears. The relative amplitude ratio (1 Kz–0.5 kHz) was significantly lower in the affected ear and in the case of the oVEMP response depends on the degree of EH. The response in the unaffected ear was not modified by the presence or the degree of hydrops.

**Conclusion:**

In the unaffected ear, hydrops is not associated with hearing deterioration. For a similar degree of hydrops, hearing loss is significantly greater in the affected ear. The endolymphatic hydrops in the vestibule induces a frequency bias in the VEMP response only in the affected ear and not in the unaffected ear. Because of these findings we consider that hydrops does not represent an active disorder in the unaffected ear.

## Introduction

The vestibular evaluation of patients with any type of dizziness such as in Ménière’s disease (MD) has undergone a major change since the introduction of new tests that analyze the reflexive response to sudden angular movements as in the video head-impulse test (vHIT) or to low sounds, skull vibrations or galvanic stimulation as in vestibular-evoked myogenic potential (VEMP).

VEMP can be recorded below the eye as close as possible to the inferior oblique muscle (ocular VEMP, oVEMP) or on the surface of the sternocleidomastoid muscle (cervical VEMP, cVEMP); the former gives the response mainly from the utricle of the contralateral side and the later mainly from the saccule of the ipsilateral side.

In patients with MD, the VEMP response depends on certain characteristics of the disease [1, 2] and of the test methodology [3]. In cVEMPs that depends also on the test frequency: there is an increase in the threshold of the response for the low frequency (0.5 kHz) or “altered frequency” tuning [4]. The abnormal response to different frequencies and the normalized p13–n23 amplitude and VEMP inhibition depth have been considered a good marker of Ménière`s disease for the detection of suspected asymptomatic hydrops in the saccule [5]. This finding has also been obtained to a lesser degree in the unaffected ear of a small group of patients with unilateral MD [6].

The difference in the tuning properties of patients with unilateral MD has also been shown when the amplitude of the response is the variable in study [7]: the amplitude of the response to 0.5 kHz is lower than expected [8], when the relative value of amplitudes obtained at 0.5 kHz and 1 kHz (cVEMP_AR0.5/1_) were considered [9]. It is a good indicator of a recent attack of vertigo as the response becomes more abnormal in comparison with that found in patients who are stable or without a documented attack close to the day of testing [10]. Frequency tuning modification and absent response are also effective in detecting endolymphatic hydrops (EH) by means of magnetic resonance imaging (MRI) in patients with MD [11].

In this work, we shall analyze the (air-conducted VEMP) AC-VEMP response in both the affected and unaffected ears of patients with unilateral MD. We shall study cervical as well ocular VEMP given also that the information in the later type of VEMP is scarce. The hypothesis is that considering the VEMP_AR0.5/1_ reduction as an indication of an abnormal function in the inner ear of patients with MD, the finding of hydrops in the unaffected ear will have functional relevance if that shifting in the tuning properties of the VEMP is also found.

## Materials and methods

### Patients

#### Inclusion criteria

The patients in this study were diagnosed with unilateral MD and fulfilled the criteria to be considered as “definite” according to the latest criteria [12]. None of the patients had been previously treated with intratympanic medication or surgically. The auditory function and vestibular tests were performed the same day and within one week from the MRI. All were followed up one year after imaging by means of clinical, auditory, and vestibular testing to rule out bilateral involvement.

#### Exclusion criteria

VEMP: when the latency of any of the wave components (p13 or n23 in the cVEMP or n10 or p16 in the oVEMP) was outside the expected interval in either of the evaluated ears [3].

Demographic data included age, sex, duration of the disease (years since the first typical episode), number of vertigo crises in the 6 months before evaluation (N) and activity of the disease, defined as days since the most recent typical vertigo crisis. Bedside vestibular examination included ocular motility, bedside VOR test, and nystagmus. Since no novel or exceptional interventions were performed in this retrospective database study, only the approval of the local ethical committee from the ENT department of the institution was required in accordance with applicable state laws. The present study was conducted in accordance with the tenets of the Declaration of Helsinki. All patients gave written consent before participating.

### Audiometry

Audiometric testing (Audiotest, Equinox IEC 645-1/ANSI S3.6-1996 type I, IEC 645-2/ANSI S3.6-1996 type B, Denmark) was performed for frequencies 0.25, 0.5, 1, 2, 3, 4 and 6 kHz under headphones. Findings of audiometry were reported in terms of the mean pure-tone average (PTA) for frequencies 0.5–3 kHz (PTA_0.5–3_) (Committee on Hearing and Equilibrium, 1995) [13], low frequency (0.25 kHz) and high frequency (mean threshold for 4, 6 kHz) or PTA_4–6_.

### VOR evaluation

This was performed with a video system (vHIT GN Otometrics, Denmark). The parameter evaluated was the VOR mean gain for head impulses on the affected (Gaff) and unaffected side (Gnaff).

### VEMP testing

VEMP response. The response evoked by cVEMP describes a positive (p13) and negative (n23) wave. In oVEMP, the response presents a negative (n10) and positive (p16) wave. The VEMPs were registered with the ICS (Chartr, Otometrics, Taastrup, Denmark) according to previously described methodology [3].

VEMP calculation of amplitude. The number of recordings made per subject was based on the reproducibility of the observed response. In those cases in which the response was absent, the mean amplitude was considered null (0 µV). To calculate the interaural asymmetry ratio (IAAR), the mean null values were artificially set at 1 µV, as in described previous work [14].

Calculation of the IAAR. It was calculated in accordance with the following formula:

$$\mathrm{IAAR}=(\frac{\mathrm{unaffected\; ear \;amplitude}-\mathrm{affected \;ear\; amplitude}}{\mathrm{unaffected \;ear \;amplitude}+\mathrm{affected \;ear \;amplitude}})\times 100.$$

Calculation of the 0.5/1 kHz amplitude ratio (VEMP_AR0.5/1_). The amplitude ratio for each ear (affected and unaffected ears) was calculated in accordance with the following formula:

$$\frac{\mathrm{VEMPAR}0.5}{1}=0.5\;\mathrm{ Khz \;amplitude}\div 1\;\mathrm{ Khz \;amplitude}.$$

**Fig. 1:**
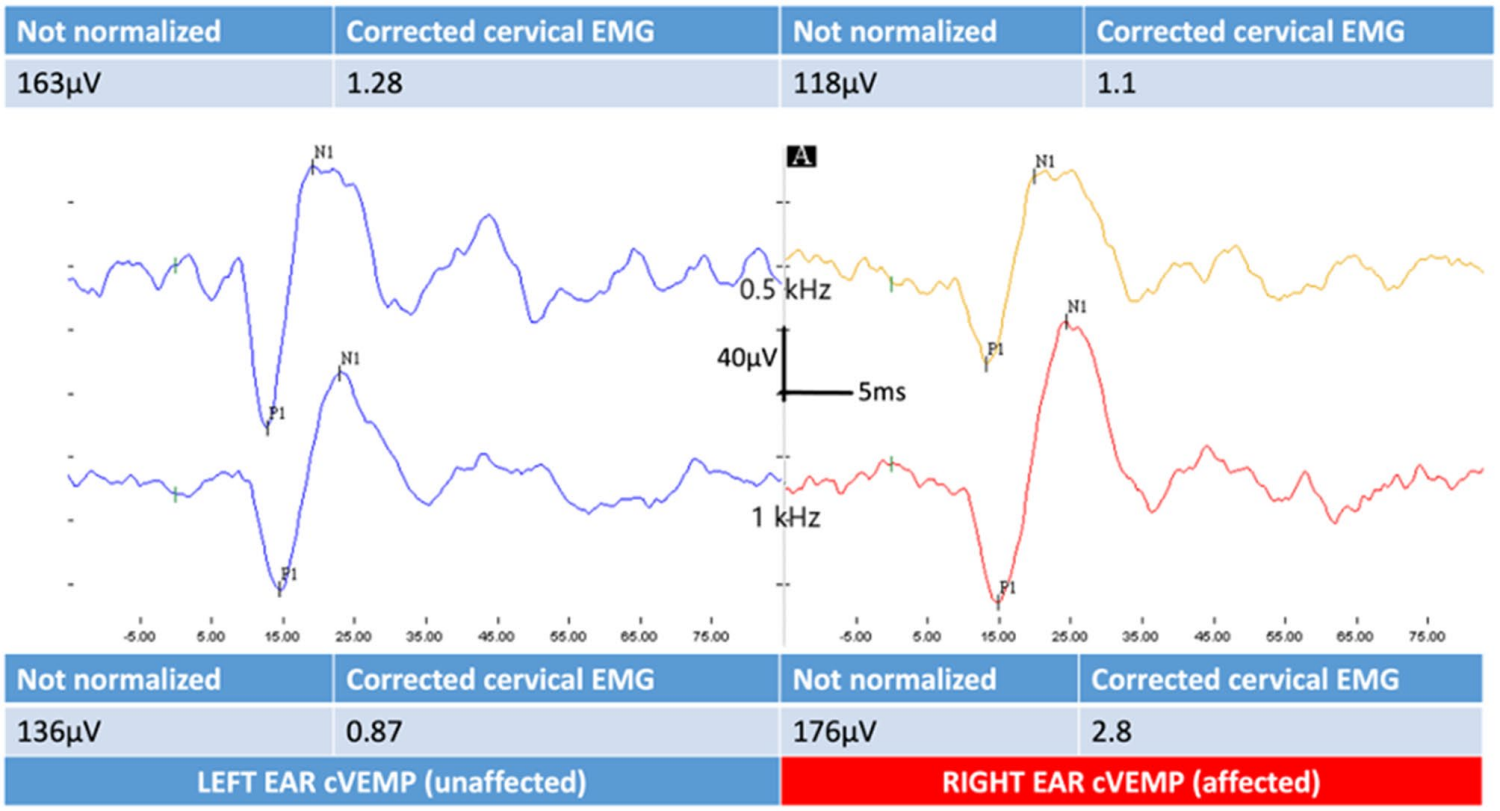
Representative cVEMP data in a 50-year-old male patient with unilateral definitive Meniere’s disease in the right ear. The affected ear shows an increased wave amplitude for the frequency of 1 kHz compared to that in 0.5 kHz. The IAAR was 7.56% for the 0.5 kHz test and − 53% for the 1 kHz test. The VEMP_AR0.5/1_ was 0.39 in the affected side and 1.47 in the left or unaffected side. cVEMP, Vestibular-evoked myogenic potential; MD, Meniere’s disease

As an example, the results in the cVEMP of a patient with a unilateral MD affecting the right ear is shown in Fig. 1.

Relative values. The IAAR (affected to unaffected response) and the 0.5/1 kHz amplitude ratio (VEMP_AR0.5/1_) in the affected and unaffected ears:

### Evaluation of endolymphatic hydrops with MRI

All MRI studies were performed in two 3 Tesla magnets, either a Siemens Magnetom Vida (Siemens Healthineers, Erlangen, Germany) with a dedicated Siemens 20-channel head coil or a Siemens Magnetom Skyra with a dedicated Siemens 32-channel head coil. The MRI hydrops dedicated sequence employed was the 3D Inversion Recovery with REAL reconstruction (3D REAL-IR) as described by Naganawa et al. [15]. This sequence was carefully chosen instead of the other hydrops sequence widely available, the 3D “Fluid attenuated inversion recovery” (FLAIR) [16]. For anatomical purposes, a heavily T2-weighted cisternography sequence was also obtained. Images were obtained 4 h after a single dose of intravenous Gd administration (Gadovist; Bayer-Schering Pharma, Berlin, Germany; 1.0 mmol/mL at a dose of 0.1 mmol/kg).

The whole imaging protocol took about sixteen minutes and consisted of:

A heavily T2-weighed sequence (T2 3D SPACE (Sampling Perfection with Application optimized Contrasts using different flip angle Evolution) with the following parameters: section thickness, 0.5 mm; TR, 1400 ms; TE, 152 ms; flip angle, 120°; bandwidth, 289 Hz/pixel; voxel size, 0.5 × 0.5 × 0.5; and scan time, 5 min. The 3D-IR: section thickness, 0.8 mm; TR, 16,000 ms; TE, 551 ms; TI: 2700 ms; flip angle, 140°; bandwidth, 434 Hz/pixel; voxel size, 0.5 × 0.5 × 0.8; and scan time, 11 min. Two very experienced head and neck radiologists qualitatively evaluated the MR images.

Cochlear endolymphatic hydrops (EH) was qualitatively assessed using a three-grade scale (none, moderate, severe) with an axial plane at a midmodiolar level [17]. For the evaluation of vestibular EH a four-grade scale was employed (none, slight, moderate, severe) [18, 19].

### Statistics

To compare the amplitudes and VEMP_AR0.5/1_ between the groups, parametric and non-parametric tests were used. The normality of the quantitative variables was studied with the Shapiro–Wilk Test. The non-parametric Kruskal–Wallis test for comparison between three and four groups was used. The correlation was calculated using the Spearman rank correlation coefficient for non-parametric variables. The descriptive statistic is expressed as median (p25, p75). All of the statistical analyses were performed with Stata 12 (StataCorp, College Station, TX).

## Results

In this work, we have included 76 patients, of which 39 (51%) were women and 37 (49%) were men. The right ear was affected in 30 patients and the left in 46. Mean age was 55 years (28–75), mean disease duration was 5 years [95% Confidence interval (CI 95) 3.7–6.6], mean number of days since the last vertigo spell was 42 (CI 95 24–60) and the mean number of vertigo spells in the previous 6 months was 6 (CI 95 5–7). The mean PTA_0.5–3_ was in the affected ear 49 ± 21 dB and in the unaffected ear 15 ± 11 dB. The mean threshold for 250 Hz was in the affected ear 54 ± 21 dB and in the unaffected ear 15 ± 10 dB, and the mean PTA_4–6_ was in the affected ear 58 ± 23 dB and in the unaffected ear 32 ± 34 dB. On vestibular examination, spontaneous nystagmus was found in 28 patients and the VOR was considered abnormal (both at bedside and with vHIT evaluation) in 20 patients.

In Table 1, we present the data for the mean PTA_0.5–3_ according to the severity of hydrops. There is a clear tendency in the mean PTA_0.5–3_ to become higher as the severity of hydrops increases in the affected ear. For a similar degree of cochlear hydrops, the PTA_0.5–3_ is significantly higher in the affected ear (for moderate severity the statistical assessment is invalid); the same occurs for vestibular hydrops (for severe hydrops also the statistical assessment is invalid).

**Table 1:** Mean pure-tone average (0.5, 1, 2 and 3 kHz) in the affected and non-affected ears according to severity of hydrops in the complete group (*N* = 76)

	*N*	Affected	*N*	Non-affected	Test and *p* value
Cochlea					
No Hydrops	14	36.66 ± 26.19	66	12.75 (8:20)	†*p = 0.015 (95% CI − 31.25:− 3)
Mild	25	41.83 ± 17.33	9	6.25 (3.3:11.6)	†*p = 0.003 (95% CI − 42.5:− 14.5)
Moderate	37	58.7 ± 16.76	1	16.25 (16.25:16.25)	††*p* = 0.979
Vestibule					
No Hydrops	9	21 (17.5:37.5)	58	12.75 (7.5:20)	†*p = 0.019 (95% CI − 26:− 1.5)
Mild	11	33.5 (23:38)	11	11.25 (8.3:31.25)	†*p = 0.045 (95% CI − 27.95: − 1.25)
Moderate	30	50.63 (36.26:63.3)	6	13.93 (10:21.25)	‡*p = 0.001 (95% CI − 49.27: − 15.09)
Severe	26	65 (55:72.5)	1	6.25 (6.25:6.25)	††*p* = 0.909

Data are expressed as p50 (p25:75), SD (±)

*Significant difference (*p* < 0.05)

^†^Wilcoxon–Mann–Whitney test; ^††^Median test; ^‡^Student test

After performing VEMP testing and according to inclusion criteria, oVEMPs were considered for evaluation in 57 patients and cVEMPs in 61. In the former group and in the affected ear, hydrops was seen in the cochlea of 43/57 and in the vestibule of 49/57; while in the unaffected ear, these data were in the cochlea and vestibule 6/57 and 12/57, respectively. In the second group (those with recognized response in both ears) and in the affected ear, hydrops was seen in the cochlea of 47/61 and in the vestibule in 53/61; while in the unaffected ear, these data in the cochlea and vestibule were 7/60 and 11/60, respectively. The proportion of hydrops in the cochlea and vestibule was not significantly different in the patients with recognized oVEMPs or cVEMPs both in the affected and unaffected ears.

In Table 2, we present the mean data of the VEMP response in the affected and unaffected ears: the amplitude of the response at 0.5 kHz, at 1 kHz, and the relative value of their amplitudes. Differences were significant for the three measures (lower amplitude of the response and VEMP_AR0.5/1_ in the affected ear) in the case of the oVEMP and only for the 0.5 kHz for the cVEMP. It is interesting to note that the mean IAAR is far from abnormal regarding our database of normal subjects.

**Table 2:** Amplitude of the response in both the affected and non-affected ears and interaural asymmetry ratio: in all cases the Wilcoxon–Mann–Whitney test was performed

	Affected ear	Non-affected ear	*p* value	IAAR (%)
cVEMP				
0.5	0.47 (0.25:0.97)	0.83 (0.32:1.45)	*p = 0.002 (CI 95% − 0.42:− 0.08)	17.24 ± 38.4
1	0.42 (0.25:0.88)	0.54 (0.28:1.14)	*p = 0.063	7.36 ± 36.64
cVEMP_AR0.5/1_	1 (0.68:1.94)	1.36 (0.84:1.94)	*p* = 0.179	
oVEMP				
0.5	1.37 (0.86:2.66)	2.68 (1.32:4.09)	*p = < 0.001 (CI 95% − 1.82:− 0.37)	22.32 ± 39.58
1	1.44 (1:2.96)	1.79 (1.11:3.45)	*p = 0.019 (CI 95% − 1.31: − 0.10)	12.3 ± 37.63
oVEMP_AR0.5/1_	0.86 (0.5:1.65)	1.18 (0.85:1.85)	*p = < 0.001 (CI 95% − 0.73: − 0.21)	

Data are expressed as p50 (p25:75), SD (±)

*Significant difference (*p* < 0.05)

The relevance of hydrops in each ear (with and without hydrops) was evaluated with the VEMP_AR0.5/1_. A significant difference was only obtained in the case of cVEMP and cochlear hydrops as shown in Table 3, which indicates that there is no frequency bias.

**Table 3:** Relative amplitude values VEMP_AR0.5/1_ according to the type of hydrops in the affected and unaffected ear

	*N*	Unaffected		Affected	Difference: test; *p* value
oVEMP					
Cochlear Hidrops	6	0.85 (0.79:3.22)	43	0.78 (0.45:1.37)	Median test; *p* = 0.701
Vestibular Hidrops	12	1.46 (0.78:2.69)	49	0.79 (0.49:1.42)	Wilcoxon–Mann–Whitney test; *p* = 0.341
cVEMP					
Cochlear Hidrops	7	1.82 (1:2.05	47	0.78 (0.67:1.62)	Median test; *p* = 0.05
Vestibular Hidrops	11	1.82 (1.36:2.3)	53	0.97 (0.67:1.93)	Median test; *p* = 0.217

**Fig. 2:**
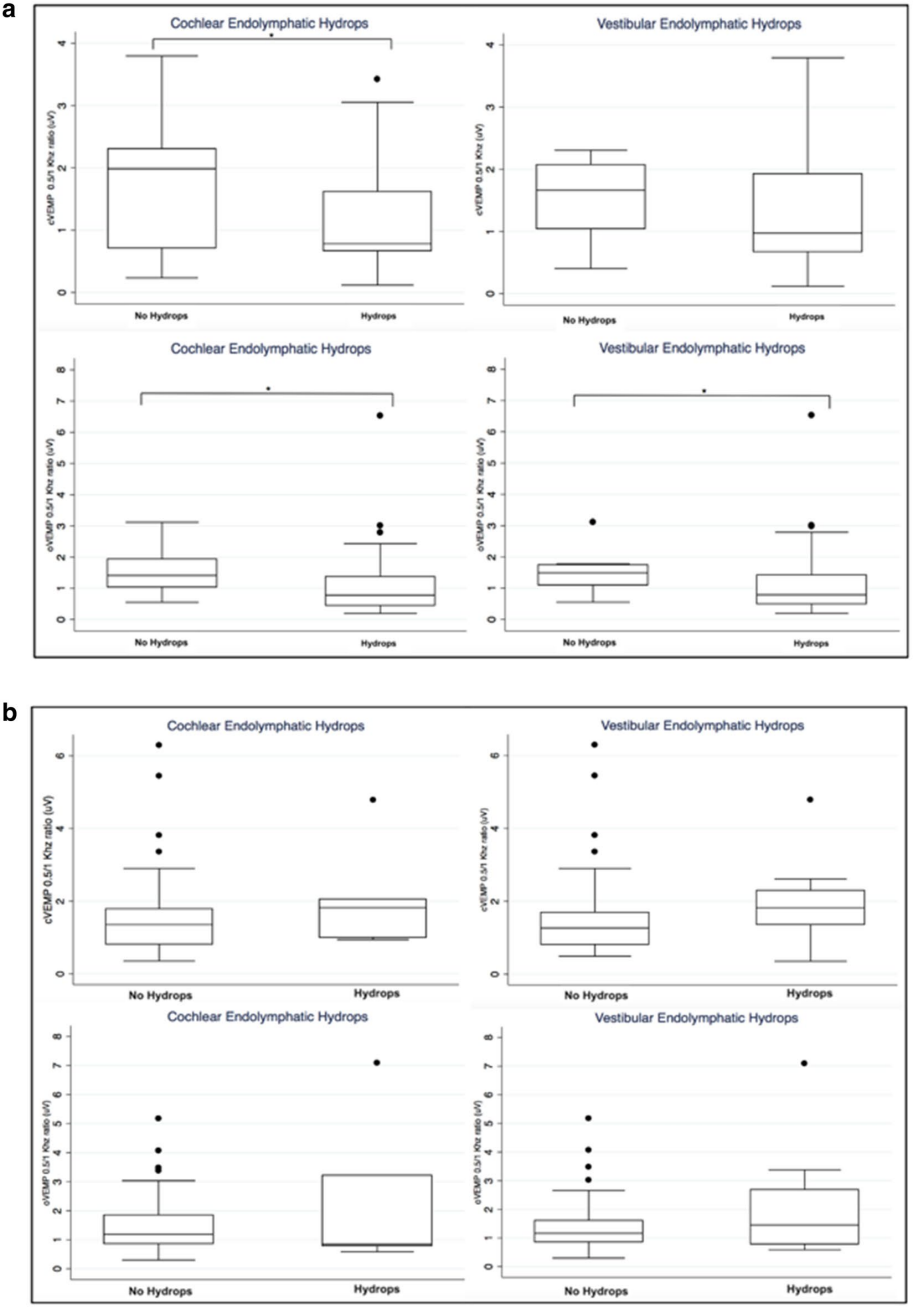
**a** The oVEMPAR0.5/1 according to the presence of cochlear and vestibular hydrops in the affected ear. Asterisks indicate significant differences (*p < 0.05). **b** The oVEMPAR0.5/1 according to the presence of cochlear and vestibular hydrops in the unaffected ear. Asterisks indicate significant differences (*p < 0.05)

In Table 4 and Fig. 2, we present the data of the VEMP_AR0.5/1_ in the affected (Fig. 2a) and unaffected (Fig. 2b) ears. As expected in the affected ear, hydrops induces a significant dysfunction as shown by the differences when hydrops was detected except for cVEMP and vestibular hydrops. However, in the unaffected ear, the VEMP_AR0.5/1_ is not significantly different whether hydrops was detected or not.

**Table 4:** The oVEMP_AR0.5/1_ and cVEMP_AR0.5/1_ according to presence or absence of endolymphatic hydrops at the cochlea or vestibule in the unaffected and affected ears

	*N*	Endolympahtic Hydrops	*N*	No hydrops	Test and *p* value
oVEMP					
Cochlea					
Unaffected	6	0.85 (0.79:3.22)	51	1.18 (0.85:1.85)	††*p* = 0.699
Affected	43	0.78 (0.45:1.37)	14	1.41 (1.03:1.93)	†*p = 0.012(95% CI 0.13:0.97)
Vestibule					
Unaffected	12	1.46 (0.78:2.69)	45	1.17 (0.85:1.62)	††*p* = 0.694
Affected	49	0.79 (0.49:1.42)	8	1.49 (1.09:1.74)	††*p = 0.003(95% CI 0.24:1.17)
cVEMP					
Cochlea					
Unaffected	7	1.82 (1:2.05	53	1.35 (0.81:1.79)	††*p* = 0.189
Affected	47	0.78 (0.67:1.62)	14	1.99 (0.71:2.31)	†*p = 0.041(95% CI 0.01:1.33)
Vestibule					
Unaffected	11	1.82 (1.36:2.3)	49	1.26 (0.81:1.69)	†*p* = 0.063
Affected	53	0.97 (0.67:1.93)	8	1.66 (1.04:2.07)	††*p* = 0.198

Data are expressed as p50 (p25:75)

*Significant difference (*p* < 0.05)

^†^Wilcoxon–Mann–Whitney test; ^††^Median test

**Fig. 3:**
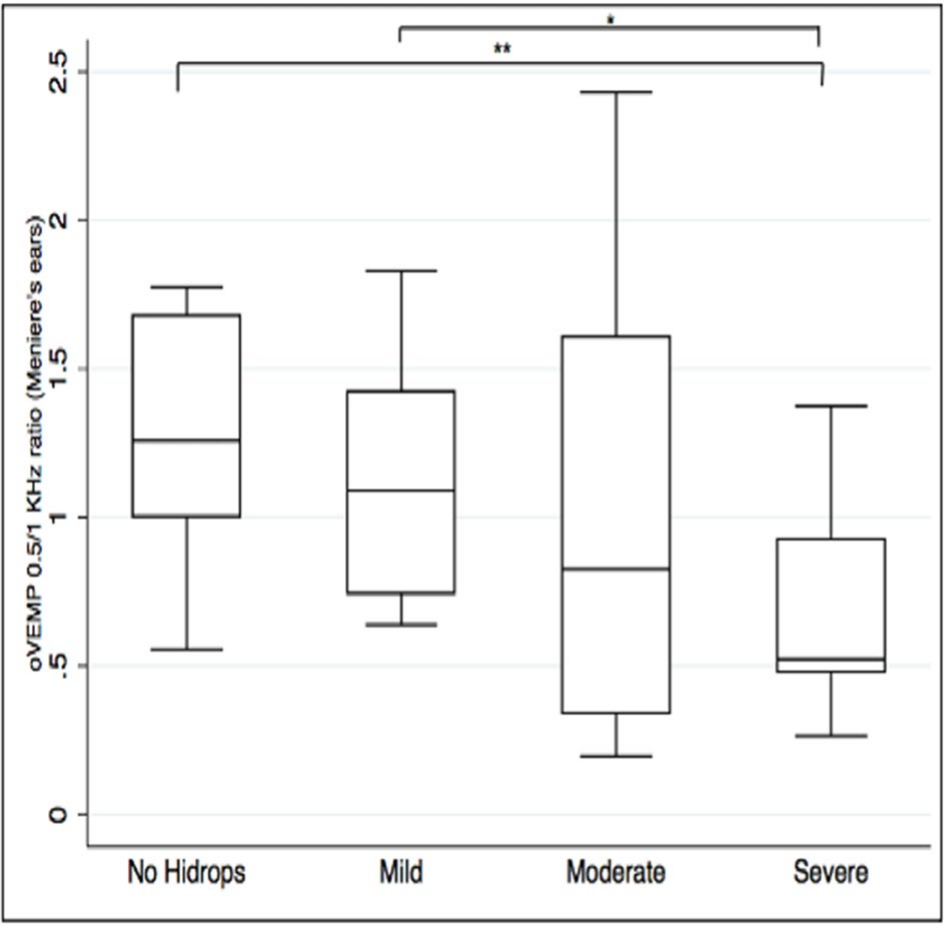
The oVEMPAR0.5/1 according to the severity of vestibular hydrops in the affected ear. Asterisks indicate significant differences (*p < 0.05, **p < 0.01)

The result in the oVEMP_AR0.5/1_ was evaluated in more detail in the case of the affected ear and, as shown in Fig. 3, we observe how this becomes lower as the severity of hydrops increases.

## Discussion

The motivation for this study came first from the consideration of four possible scenarios that we commonly face at present when dealing with patients with “definite” unilateral MD after MRI evaluation disease. The most common scenario is when cochlear or vestibular hydrops are detected in the MRI only in the affected ear as occurs in 70% of the patients in our study. The second is when hydrops is detected in both ears owing to simultaneous cochlear or vestibular EH as seen in 25% of our patients. The third and fourth scenarios (EH only in the unaffected ear, or no hydrops in either ear) are markedly unexpected: 1% and 4%, respectively.

### EH in the affected ear

The amount of hydrops in the affected ear of patients with unilateral MD is in the higher range of what has been reported by previous authors [20], but, however, is similar to what has been reported in otopathology reports. Our 94% EH detection, when both cochlea and vestibule are considered, has two main reasons: the population under study and the technique itself. Our population was made up only of patients who fulfilled the criteria for “definite” unilateral MD according to the most recent criteria and were very homogeneous in terms of disease duration. This can be considered as medium and is important because it could influence the severity of hydrops as seen in the MRI: longer disease duration is associated with more severe EH [21], although there are reports that do not agree on this association [22]. As a limitation to our study, the precise characteristics of initiation of the disease was not noted. In the case of recent onset disease, the clinical presentation (which differs very much between patients) is probably another source of variability. It has been shown that in 61% patients, auditory symptoms occur first (months before the first vertigo crisis) and complete (auditory plus vestibular) after, but that the opposite (vestibular first) occurs in 18%; both appear simultaneously in 21% [23]. The second cause of our high detection rate is that EH with MRI can be over-diagnosed according to reports in normal subjects [24].

### EH in the unaffected ear

Hydrops in the unaffected ear was found in 20/76 (26.3%) of patients and was more frequent in the vestibule: in seven patients, hydrops was found both in the cochlea and vestibule, in three in the cochlea and in 10 only in the vestibule. In one patient, hydrops was only seen in the unaffected ear: this is a 50-year-old female with a history of MD in her left ear of one year duration when the MRI was performed; she was also diagnosed with migraine with aura, but the last attack of migraine took place almost 1 year before MRI. It is well known that EH is mostly related to cochlear dysfunction in cases of vestibular migraine with auditory symptoms which is related to the degree of cochlear or vestibular hydrops [25].

The number of patients with hydrops in the unaffected ear is very similar to that reported by others using MRI to detect EH and it considered to be part of a more severe disease or with a longer duration [26]. Is also similar to the number of patients expected to develop bilateral MD [27–30]. This is by no means a consistent argument when deciding that a particular technique showing that number could eventually identify potential bilateral MD patients in advance, mainly when considering the extreme differences in studies addressing the incidence of bilateral MD [7]. Also, we have to take into account that 20% of patients with unilateral MD show EH in the unaffected ear at postmortem examination [31].

In our study, we were, therefore, interested in analyzing hearing and vestibular function: the former by means of audiometric findings and the later with AC-VEMP. And the question was whether EH in the contralateral also indicates auditory or vestibular dysfunction in that “normal” ear.

Here, we have shown that for a similar degree of hydrops in both ears, there are significant differences in the amount of hearing damage: the PTA is higher in the affected ear when cochlear and vestibular hydrops were mild and when vestibular hydrops was moderate. In the other case of moderate cochlear hydrops, there were not enough unaffected ears as to statistically compare results. In the case of “no hydrops” at all in both ears, there are also differences in the PTA that continues to be significantly higher in the affected ear. This can be explained by different hypotheses. In the first case, the inability to detect subtle changes in the cochlea (of the unaffected ear) with current methodology [32]. Use of the intratympanic route for gadolinium administration [33] or electrocochleography could both be methods to better analyze that situation and better characterize those ears [34]. In second place, absent EH in the affected ear with abnormal hearing could also indicate that hydrops is not the only relevant change to symptomatology [35] as we know occurs in the contrary, well-developed disease [36].

Inclusion and exclusion criteria were set so as to have patients with consistent responses or VEMPs and for this reason, the initial number of patients was reduced. The patients who were excluded did not share any specific characteristic in terms of clinical parameters. The severity of hydrops was also randomly distributed in that group. The number of patients now under study probably explains that some results were not congruent [37]. As expected, and in accordance with previous mentioned findings, we found lower VEMP_AR0.5/1_ in the affected ear as compared to the unaffected ear [38]. This difference between ears was significant in the case of the oVEMP but not with cVEMP. As shown in Table 2, this is because the amplitude of the response in the affected ear was significantly lower than in the unaffected ear for the 0.5 kHz stimulus but not for the 1 kHz in cVEMP[8]. In the case of the oVEMP, the same occurred, but the amount of amplitude difference between the affected and unaffected ears for the 1 kHz stimulus was lower in proportion but becoming significant. This paradoxical behavior needs to be corroborated also with bone-conducted stimulus and in larger studies because it does not match the well-known data form experimental work on saccular afferents threshold [39].

With the possibility of analyzing results in accordance with the degree of EH, we show that when hydrops is found in the cochlea, there is a more severe dysfunction in the affected ear as indicated by frequency tuning. In the case of hydrops in the unaffected ear, we have not found EH to be related to significant differences in the value of the 0.5/1 kHz amplitude ratio. For this reason, we consider that, when dealing with patients with unilateral MD, VEMP testing must be part of the laboratory evaluation given its ability to detect more subtle changes in EH [40].

These findings must not be overlooked and should be integrated into the final decision on treatment with patients who are not doing well, and when an ablative or semi-ablative treatment is considered for the affected ear.

## Conclusions

EH occurs in patients with unilateral MD more frequently only in the affected ear but can also be found in both ears.

In the unaffected ears hydrops is not associated with hearing deterioration: for a similar degree of hydrops in the affected and unaffected ear, hearing loss is significantly greater in the former.

The amount of vestibular dysfunction as shown by the 0.5/1 kHz amplitude ratio needs to be part of the evaluation during follow-up to better acknowledge its relevance in EH development in the unaffected ear.
